# The Immature Fiber Mutant Phenotype of Cotton (*Gossypium hirsutum*) Is Linked to a 22-bp Frame-Shift Deletion in a Mitochondria Targeted Pentatricopeptide Repeat Gene

**DOI:** 10.1534/g3.116.027649

**Published:** 2016-03-29

**Authors:** Gregory N. Thyssen, David D. Fang, Linghe Zeng, Xianliang Song, Christopher D. Delhom, Tracy L. Condon, Ping Li, Hee Jin Kim

**Affiliations:** * Cotton Fiber Bioscience Research Unit, USDA-ARS-SRRC, New Orleans, Louisiana 70124,; † Cotton Chemistry and Utilization Unit, USDA-ARS-SRRC, New Orleans, Louisiana 70124,; ‡ Crop Genetics Research Unit, USDA-ARS, Stoneville, Mississippi 38776,; § College of Agronomy, State Key Laboratory of Crop Biology, Shandong Agricultural University, Shandong, China; ** Cotton Structure and Quality Research Unit, USDA-ARS-SRRC, New Orleans, Louisiana 70124

**Keywords:** Cotton fiber cell wall thickness, fiber maturity, frame-shift deletion, *im* locus, pentatricopeptide repeat (PPR)

## Abstract

Cotton seed trichomes are the most important source of natural fibers globally. The major fiber thickness properties influence the price of the raw material, and the quality of the finished product. The recessive immature fiber (*im*) gene reduces the degree of fiber cell wall thickening by a process that was previously shown to involve mitochondrial function in allotetraploid *Gossypium hirsutum*. Here, we present the fine genetic mapping of the *im* locus, gene expression analysis of annotated proteins near the locus, and association analysis of the linked markers. Mapping-by-sequencing identified a 22-bp deletion in a pentatricopeptide repeat (PPR) gene that is completely linked to the immature fiber phenotype in 2837 F_2_ plants, and is absent from all 163 cultivated varieties tested, although other closely linked marker polymorphisms are prevalent in the diversity panel. This frame-shift mutation results in a transcript with two long open reading frames: one containing the N-terminal transit peptide that targets mitochondria, the other containing only the RNA-binding PPR domains, suggesting that a functional PPR protein cannot be targeted to mitochondria in the *im* mutant. Taken together, these results suggest that PPR gene Gh_A03G0489 is involved in the cotton fiber wall thickening process, and is a promising candidate gene at the *im* locus. Our findings expand our understanding of the molecular mechanisms that modulate cotton fiber fineness and maturity, and may facilitate the development of cotton varieties with superior fiber attributes.

Cotton is the world’s most important source of natural fibers for textiles. Cotton breeders have long faced the challenge of simultaneously improving fiber quality and yield (Clement *et al.* 2014). Among the major fiber properties are thickness-related properties including fineness and maturity, which affect the quality of the produced yarn. Finer fibers allow for more fibers per cross section of yarn, improving yarn tenacity, and delivering a finer yarn for high end garments (Clement *et al.* 2014). Fiber maturity affects the ability of the yarn to be dyed, and is a measure of the degree of thickening of the cotton fiber cell wall (Bradow *et al.* 1996). Cotton breeders have found that fiber quality is generally negatively correlated with yield, so a deeper understanding of the genetic mechanisms that control these traits may enable a decoupling of this correlation.

The immature fiber mutant was identified in the early 1970s and is used as a model to understand the development of cotton fiber cells (Kohel *et al.* 1974). This mutant was identified by thin fibers with reduced cell wall thickening, resulting in nonfluffy bolls of mature cotton. The immature fiber was caused by a single recessive gene, designated *im*, that was shown to reside on chromosome 3 by aneuploidy tests and genetic marker analysis (Kim *et al.* 2013a; Kohel *et al.* 2002; Wang *et al.* 2013). Analysis of transcription during fiber development in *im* plants, along with near-isogenic wild-type plants, suggested roles for cell wall, stress response, and respiratory genes in the generation of the mutant phenotype (Kim *et al.* 2013b; Wang *et al.* 2014). Interestingly, the identification of altered mitochondrial oxidase pathways successfully predicted differences in reactive oxygen species that were also observed in developing *im* fibers, supporting a key role for the mitochondria in the development of mature cotton fiber cells (Kim *et al.* 2013b).

Recently, the release of draft and reference genomes for *Gossypium* species have accelerated candidate gene discovery for major genes in cotton by mapping-by-sequencing (Thyssen *et al.* 2014a, 2015). The insertion of a retrotransposon into a homeodomain transcription factor has been proposed to underlie the T1 dominant stem trichome gene (Ding *et al.* 2015). Another striking mutation affecting the protein sequence of a different homeodomain protein has been linked to the okra leaf phenotype in cotton (Zhu *et al.* 2015). In this study, we use mapping-by-sequencing, and a newly released draft *G. hirsutum* genome, to identify a striking 22-bp deletion in a cotton ortholog of a mitochondria targeted pentatricopeptide repeat (PPR) gene (Zhang *et al.* 2015). This deletion results in a frame shift, which abolishes the ability for the transcript to encode a functional full length protein that contains both the mitochondria-targeting transit peptide and the RNA-binding PPR domains. We found that this deletion is completely linked to the *im* gene in 2837 F_2_ plants. Importantly, we also found that it is absent from 163 cultivated wild-type varieties that produce thick and mature fibers, although nearby marker polymorphisms are prevalent in the diversity panel. Therefore, we propose PPR gene Gh_A03G0489 as a candidate gene at the *im* locus. We expect that alternative alleles of this gene will be useful for developing cotton varieties with superior fiber properties.

## Materials and Methods

### Plant materials

The plant materials used in this study comprised: 163 cultivated accessions of *G. hirsutum* in a diversity panel (Supplemental Material, Table S1), and three F_2_ populations segregating for the immature fiber trait along with their parent lines. The first F_2_ population (Population 1) was described previously and contained 366 plants (270 wild type: 80 *im*: 16 no phenotype) that were derived from a cross between *G. hirsutum* cultivar TM-1 and its near isogenic line (NIL) containing the *im* gene (Kim *et al.* 2013a). The second F_2_ population (Population 2) had the same parents, contained 1880 plants (1299 wild type: 469 *im*: 112 no phenotype), and was planted in 2013 in a field in Stoneville, MS. The third F_2_ population (Population 3) derived from a cross between the *G. hirsutum* cultivar MD 52ne and *im* mutant, and contained 735 plants (560 wild type: 159 *im*: 16 no phenotype). This population was grown in a field in Stoneville, MS in 2014. Naturally opened bolls were hand harvested and ginned using a laboratory roller gin. Phenotypes were scored based on lint percentage as before (Kim *et al.* 2013a). Micronaire (MIC) data were also measured using a high-volume instrument (HVI) for all the plants in Population 3. For Populations 1 and 2, MIC data were measured only for the plants that had marginal lint percentages (in the range 26–29%). Generally, MIC values below 3.5 were considered immature phenotype. Parental *im* and TM-1 NILs were grown in a field in New Orleans, LA in 2013 for mRNA isolation. Standard conventional field practices were followed at both locations and in all years. DNA was isolated from young leaves as described previously (Fang *et al.* 2010).

### RNA isolation, RNAseq, and RT-qPCR

Three biological replicates of fibers from different developmental time points were collected from the field in New Orleans, LA. Total RNA from 28-d post anthesis (DPA) fiber cells from two replicates were Illumina sequenced by Data2Bio LLC (Ames, IA) with paired-end 101-bp reads. Total RNA from the three biological replicates of 10, 17, and 28-DPA fiber cells were converted to cDNA, and subjected to reverse transcription quantitative polymerase chain reaction (RT-qPCR) as described elsewhere (Naoumkina *et al.* 2015). Primer sequences for RT-qPCR are provided in Table S2. RNAseq gene expression was quantified using GSNAP and bedtools software, and differential gene expression was evaluated by trimmed mean of M (TMM) normalization and ANOVA analysis as before (Naoumkina *et al.* 2014). We calculated and present RPKM normalized expression values, although we present *P*-values derived from the TMM analysis (Mortazavi *et al.* 2008).

### Super bulked segregant analysis sequencing (sBSAseq)

F_2_ plants from Population 1 were selected for sequencing by a bulked segregant analysis approach (Michelmore *et al.* 1991; Takagi *et al.* 2013). Two DNA pools were constructed: a pool of DNA from 80 *im* fiber and a pool of DNA from 80 wild-type plants. DNA was Illumina sequenced with paired-end 101-bp reads by BGI Americas (Cambridge, MA).

### Mapping-by-sequencing, marker design, and fine mapping the im locus

We aligned the sBSAseq total genomic reads, using GSNAP software, to a draft reference genome for *G. hirsutum* cultivar TM-1 (Wu and Nacu 2010; Zhang *et al.* 2015). We used vcftools software to call SNPs and indels between the wild type and mutant bulk sequences (Danecek *et al.* 2011). We generated a histogram by counting the number of SNPs with quality > 50 in 10-Mb intervals. We manually inspected the alignment files to confirm indels in the vicinity of the *im* locus on chromosome 3, which corresponds to chromosome A03 in the reference genome (Kim *et al.* 2013a; Zhang *et al.* 2015). We extracted all reads that mapped to D subgenome chromosomes and realigned them to a reference sequence that contained only the A subgenome chromosomes so that we could identify homeoSNPs near our marker loci. We used the homeoSNPs to design subgenome specific primers essentially as described previously (Thyssen *et al.* 2014a). Each forward primer ends with the mutant allele SNP, while each reverse primer ends with the A subgenome homeoSNP. Both primers contain an additional mismatch at the third base from the 3′ end, which increases annealing temperature stringency (Drenkard *et al.* 2000). Primers flanking indels include one subgenome specific primer. The 2981 F_2_ plants were tested for segregation with the new SNP and indel markers, along with two SSR markers, DPL1071 and SHIN-1511, that were previously linked to the *im* gene and shown to reside on chromosome 3 (Kim *et al.* 2013a). We also designed primers to test one SNP from the CottonSNP63k array, i00510GH, on the F_2_ progeny (Hulse-Kemp *et al.* 2015). Primer sequences are included in Table S2. The linked indel marker CFBid0001 and three closest markers (CFB5887, CFB5888, and CFBid0002), were tested on a diversity panel of 163 accessions of *G. hirsutum*. The PCR methods for genotyping SNP markers were described previously (Thyssen *et al.* 2014a). For genotyping indel markers, the forward primers were fluorescent-labeled at 5′ end with 6-FAM (6-carboxyfluorescein), or HEX (4, 7, 2′, 4′, 5, 7-hexachloro-carboxyfluorescein). Primers were purchased from Sigma Genosys (Woodlands, TX). PCR amplification was according to Fang *et al.* (2010). Amplified PCR products were separated and measured on an automated capillary electrophoresis system ABI 3730 XL (Applied Biosystems Inc.). GeneScan-400 ROX (Applied Biosystems Inc.) was used as an internal DNA size standard. A genetic linkage map was generated with JoinMap 4.0 software using default parameters (Van Ooijen 2006).

### Transcript sequence analysis

Consensus sequences for the *im* and TM-1 alleles of Gh_A03G0489 were extracted from the RNAseq and sBSAseq alignment files with IGV software (Robinson *et al.* 2011). Open reading frames were identified using the translate tool software at ExPASy (Gasteiger *et al.* 2003). The subcellular target and cleavage site of the N-terminal transit peptide were predicted using TargetP software (Emanuelsson *et al.* 2000; Nielsen *et al.* 1997). PPR motifs were found using the PROSITE entry PS51375 and the ScanProsite software (De Castro *et al.* 2006; Sigrist *et al.* 2013). The RNA-binding site specificity of the PPR sequence was predicted following the method of Yagi *et al.* (2013). The resulting probability matrix was converted to a sequence logo by Seq2Logo software (Thomsen and Nielsen 2012).

### Data availability

All relevant data are within the paper and the associated Supplemental Material files. Table S1 contains detailed descriptions of all studied cotton accessions. Table S2 contains all of the primers designed for this research. Figure S2 contains the consensus coding sequences obtained for the *im* and TM-1 alleles of PPR Gh_A03G0489.

## Results

### The immature fiber mutant phenotype

**Figure 1:**
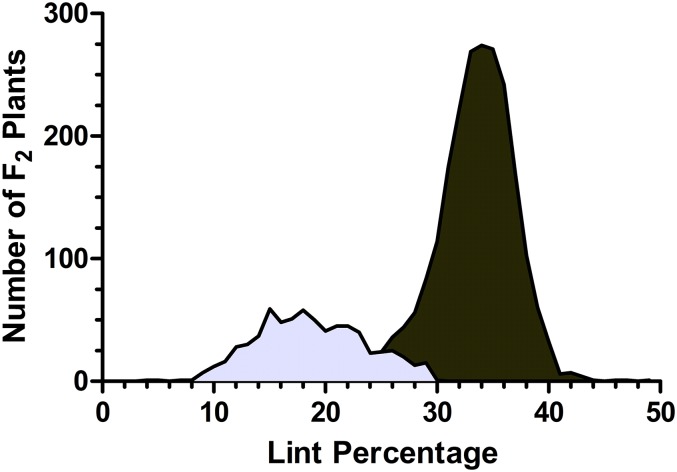
Lint percentage in 2837 F_2_ plants segregating for the immature fiber trait. Wild-type plants are represented by the dark gray histogram, while *im* plants are light gray. Plants producing less than 25% lint were scored as immature fiber, and plants with 26–29% lint were further evaluated by micronaire (MIC).

As before, we were able to treat the *im* phenotype as a qualitative character based on lint percentage and MIC (Kim *et al.* 2013a). Plants from segregating F_2_ plants were scored as wild type or *im* mutant using a threshold of 25% lint, and marginal plants were determined based on MIC value. The populations segregated 3:1, confirming that *im* phenotype is controlled by a single recessive locus (Figure 1). Comprehensive analysis of *im* fiber properties has been reported elsewhere (Kim *et al.* 2013a, 2013b).

### Mapping-by-sequencing

**Figure 2:**
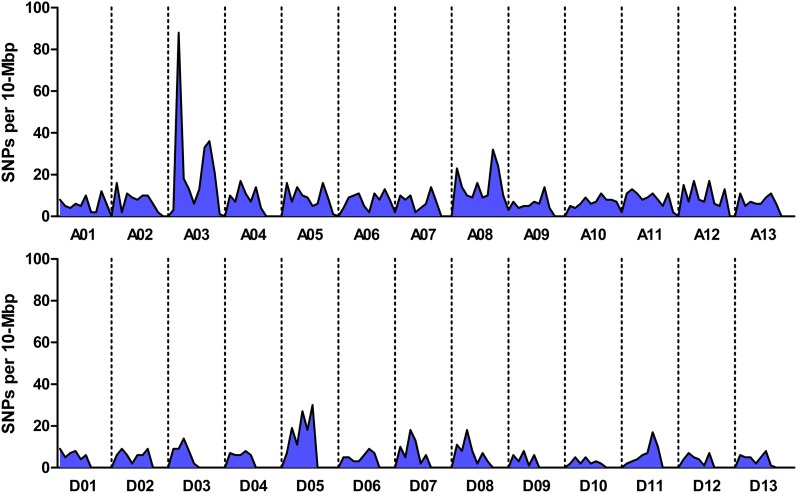
Distribution of SNPs in sBSAseq data from bulked immature and wild-type F_2_ plants. The chromosomes are named according to the draft reference *Gossypium hirsutum* cv. TM-1 genome.

**Figure 3:**
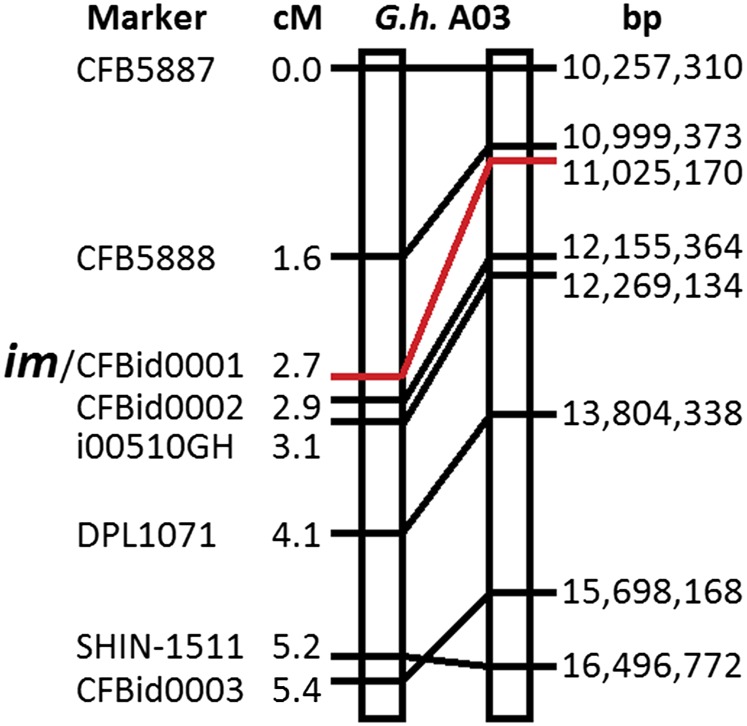
Genetic and physical map of the immature genetic locus based on 2981 F_2_ plants. Genetic distances are shown in centiMorgans (cM), and markers are also located on chromosome A03 of the draft reference *G. hirsutum* cv. TM-1 genome.

Comparison of the sequences obtained from sBSAseq clearly identified a region of chromosome 3 (Chr. A03) with high nucleotide polymorphism between the *im* mutant and wild-type bulks of F_2_ plants (Figure 2). The striking peak on Chr. A03 identified 88 high quality SNPs between the 10th and 20th Mb of the reference Chr. A03 (Zhang *et al.* 2015). This region is consistent with earlier reports, and contains sequences that match SSRs that were previously linked to the *im* gene (Figure 3) (Kim *et al.* 2013a; Wang *et al.* 2013).

### Fine linkage mapping

To explore linkage between sequence polymorphisms in the sequencing data on Chr. A03 and the *im* gene, we scored two new SNP markers (CFB5887 and CFB5888), three new indel markers (CFBid0001, CFBid0002, and CFBid0003), a SNP from the CottonSNP63k array (i00510GH), and two SSRs (DPL1071 and SHIN-1511) on 2981 segregating F_2_ plants. Although they lacked phenotypes, 144 of these plants were included to determine accurate genetic distances between the markers. Generally, our genetic markers were spaced each megabase of physical distance, and were found to be about a centiMorgan apart, giving a 1 cM/Mb ratio in this region (Figure 3). All 708 plants that displayed the *im* phenotype were homozygous for the allele of the CFBid0001 marker that is present in the *im* NIL that was a parent in each F_2_ population. Furthermore, no wild-type plant was homozygous for the *im* allele of CFB0001, indicating complete linkage of this marker with the immature fiber phenotype in 2837 F_2_ plants (Figure 3). Of the closely linked markers, only CFBid0001 is located in the coding sequence of a gene, and is a 22-bp deletion in a PPR gene, Gh_A03G0489.

### Gene expression analysis

We examined the expression of all annotated proteins between markers CFB5887 and CFBid0002, which corresponds to approximately 1-Mb of sequence on either side of the completely linked marker, CFBid0001 (Figure 3). Of the 38 annotated proteins in this interval, only 15 were detected at > 1 RPKM in either mutant or wild type fibers at 28 DPA. Of these, only one, Gh_A03G0468, which has similarity to protoheme IX farnesyltransferase, was significantly differentially expressed (*P* < 0.05) (Table 1). We tested all 38 genes for relative transcript abundance by RT-qPCR in 10, 17, and 28-DPA fiber cells, but were able to detect expression of only 28 genes. None of these genes showed significant, twofold differential expression between immature and wild-type fibers at any of the time points, including Gh_A03G0468 and the PPR gene Gh_ A03G0489 (Figure S1).

**Table 1:** Gene expression near the *im* locus in 28-DPA fiber cells by RNAseq

Gene/Marker	Position Chr. A03	TM-1 RPKM	*im* RPKM	log2 (*im*/TM-1)	*P*-value	TAIR Ortholog	Description
CFB5887	10,257,310						Flanking SNP marker
Gh_A03G0468	10,292,732	6.4	1.5	−2.1	0.024	None	Protoheme IX farnesyltransferase
Gh_A03G0469	10,294,222	1.2	1.4	0.2	0.052	AT5G50580	SUMO-activating enzyme 1B
Gh_A03G0473	10,352,804	1.5	1.3	−0.2	0.752	AT3G26890	None
Gh_A03G0475	10,381,659	3.1	2.9	−0.1	0.685	AT4G00650	FRIGIDA-like protein
Gh_A03G0480	10,491,490	1.4	1.2	−0.2	0.840	AT5G51050	Mitochondrial substrate carrier family protein
Gh_A03G0483	10,599,666	3.0	2.3	−0.4	0.193	AT5G51020	CRUMPLED LEAF
Gh_A03G0484	10,616,903	16.8	15.9	−0.1	0.660	AT1G01630	Sec14p-like phosphatidylinositol transfer protein
CFB5888	10,999,373						Flanking SNP marker
Gh_A03G0489	11,022,670	2.4	1.0	−1.3	0.164	AT1G64580	Pentatricopeptide repeat (PPR) protein, mitochondrial
CFBid0001	11,025,170						*im*-Linked 22-bp deletion in exon of PPR
Gh_A03G0491	11,449,872	12.7	9.6	−0.4	0.192	AT5G50920	Chaperone protein ClpC, chloroplastic
Gh_A03G0492	11,458,109	4.0	4.3	0.1	0.139	AT2G42490	Copper methylamine oxidase
Gh_A03G0493	11,495,867	29.6	26.1	−0.2	0.933	AT4G24690	NBR1, a selective autophagy substrate
Gh_A03G0495	11,523,884	2.9	1.8	−0.7	0.676	None	None
Gh_A03G0496	11,525,162	1.1	0.3	−1.9	0.126	AT4G24700	None
Gh_A03G0498	11,626,203	14.3	13.5	−0.1	0.457	AT4G20360	RAB GTPase homolog E1B
Gh_A03G0500	11,796,370	1.7	1.9	0.2	0.529	AT5G62350	Pectin methylesterase inhibitor superfamily protein
CFBid0002	12,155,364						Flanking deletion marker
Gh_A03G0506	12,155,624	0.0	0.0	1.6	0.559	AT1G12260	NAC 007

Only annotated proteins with >1 RPKM and a select cell wall gene are presented. Nearby marker locations are indicated

### Association of markers in diversity panel

**Figure 4:**
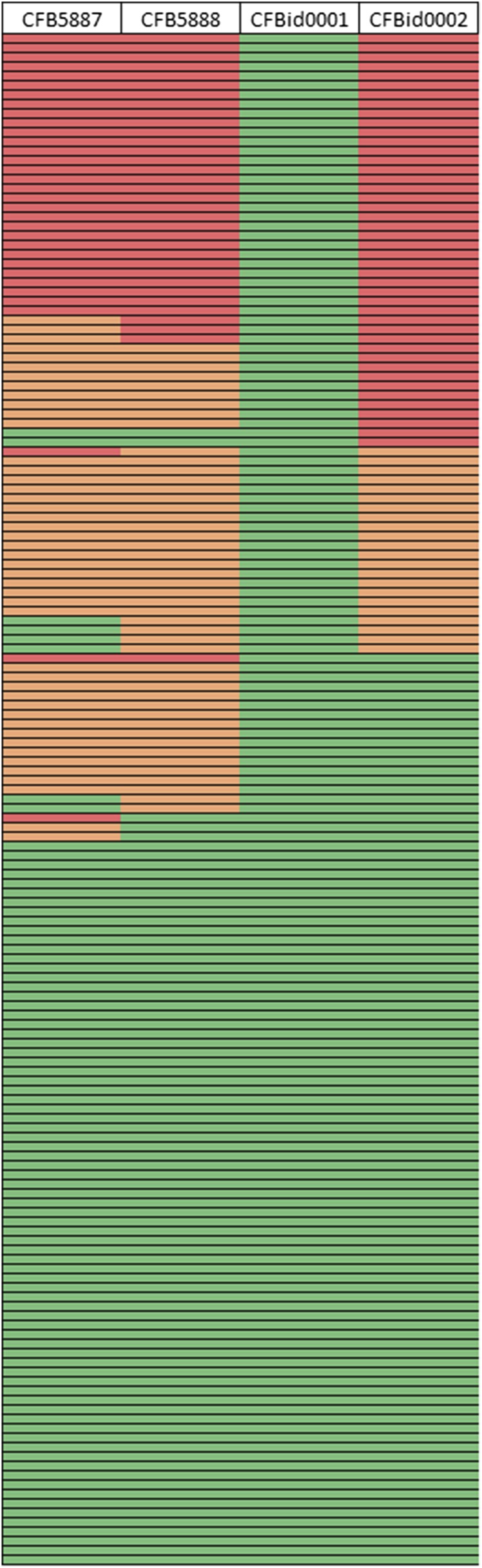
Association of closely linked genetic markers in a diversity panel of 163 accessions of *G. hirsutum*. Each row is an independent variety described in Table S1, and the columns are labeled with the respective genetic markers. Homozygocity for the *im*-type allele is shown in red, homozygocity for the TM-1-type allele is shown in green, and heterozygocity for a marker is indicated with orange.

To ascertain if any of our closely linked markers could be the causative mutation that controls the immature phenotypic trait, we tested the completely linked marker CFBid0001, and three closely linked markers (CFB5887, CFB5888, and CFBid0002) on a diversity panel of 163 cultivated wild-type varieties (Figure 4 and Table S1). Of these varieties, not one contained an *im*-type allele of CFBid0001/Gh_A03G0489, including 30 lines that were homozygous for the *im*-type allele of all three nearby markers. These 30 varieties originate from diverse countries, including the USA, China, Australia, Pakistan, India, and Uzbekistan (Table S1). An additional 56 varieties contained *im*-type alleles in at least one of the three nearby markers (Figure 4 and Table S1).

### Characterization of PPR Gh_ A03G0489 transcripts

**Figure 5:**
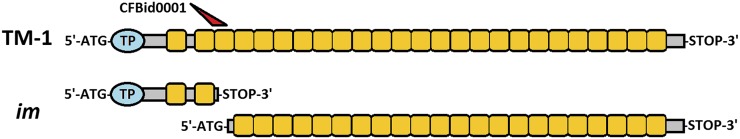
Open reading frames in immature (*im*) and wild-type (TM-1) transcripts of Gh_ A03G0489. The N-terminal mitochondrial transit peptide is labeled with TP. The location of the *im*-linked 22-bp deletion, CFBid0001, is indicated. The RNA-binding pentatricopeptide repeats (PPR) are indicated with squares with rounded corners.

**Figure 6:**
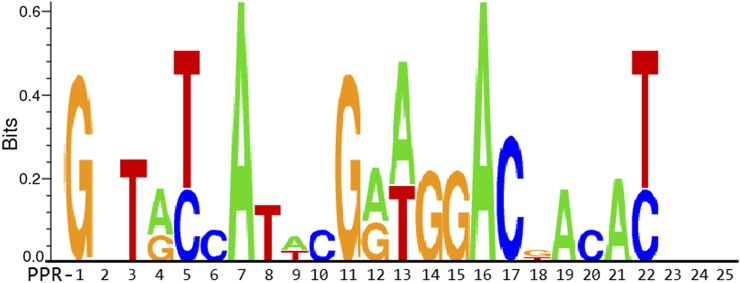
Predicted mitochondrial RNA-binding motif for PPR gene Gh_A03G0489. The nucleotide specified by each of the 25 PPR domains is shown. The height of each letter is proportional to the expected frequency of binding. For simplicity, only positive bit scores are shown. For a complete position-specific weight matrix, see Table S3.

To predict the consequences of the 22-bp deletion in PPR Gh_A03G0489, we first obtained the full-length consensus coding sequences for the *im* and TM-1 alleles from our short read RNAseq and sBSAseq alignments (Figure S2). Next, we identified open reading frames (ORFs) in each transcript. The wild-type transcript contained a single ORF that corresponded to the full-length protein sequence [1023 amino acids (aa)], as expected, while the *im* transcript contained two ORFs longer than 100 aa (Figure S3). One of these ORFs begins at the canonical ATG but, because of the frame-shift deletion, terminates after 184 aa. The second ORF initiates from a downstream ATG and contains the C-terminal 813 aa of the reference protein (Figure S3). Since the annotation for the protein identifies it as an ortholog of a mitochondria-targeted PPR protein, we used the ORFs, a reference PPR motif, and a transit peptide database to further characterize the proteins. Like its orthologs, Gh_A03G0489, has an N-terminal transit peptide that is predicted to confer mitochondrial targeting (Table S3). The full-length protein sequence contains 25 PPR-like repeats, with 24 in immediate succession (Figure 5 and Table S3). Of the two ORFs in the *im* transcript, one contains the transit peptide sequence and the first two PPR repeats, while the other long ORF contains the C-terminal 22 PPRs. Since the crystal structure of PPR proteins bound to RNA has been solved and PPR binding preferences identified, we followed the method of Yagi *et al.* (2013) to identify the critical nucleotide specifying residues in each repeat and predict the nucleic acid binding motif of wild-type Gh_A03G0489, which we present as a sequence logo and position-specific weight matrix (Figure 6 and Table S3).

## Discussion

### Mapping-by-sequencing of the immature fiber (im) gene

We took advantage of the recently released draft reference genome for *G. hirsutum* cultivar TM-1 to identify a region of diversity between two bulked pools of F_2_ plants that were segregating for the *im* gene (Figure 2). Within the 10-Mb interval that contained the highest density of SNPs, we also identified indels, which, along with the SNPs, we used to design genetic markers to test on individual F_2_ plants from a total population of 2981 (Figure 3). Of these, 2837 plants produced enough fiber to be phenotyped. One deletion marker, CFBid0001, was completely linked to the phenotype in the segregating progeny (Figure 3). This 22-bp deletion is a striking frame-shift mutation in the coding sequence of a PPR gene, Gh_A03G0489 (Figure S2 and Figure S3).

### Diversity panel

A nearby, but not completely linked, marker, CFBid0002, is a deletion less than 300 bp upstream of Gh_A03G0506, which is an ortholog of NAC 007, a transcription factor that orchestrates secondary cell wall development in *Arabidopsis* (Table 1) (Ooka *et al.* 2003; Zhou *et al.* 2014). Because of the altered secondary cell wall thickness in fiber cells of plants with the immature fiber phenotype, we also considered this mutation as an appealing candidate near the *im* locus. Since the causative mutation for the immature fiber trait results in an obvious phenotype, it should be absent from most, if not all, cultivated *G. hirsutum* varieties. When we tested the four closest markers on a panel of 163 cultivars, we found that the deletion in the NAC promoter was prevalent, but the CFBid0001 deletion in the PPR coding sequence was completely absent (Figure 4 and Table S2). Additionally, the two nearby SNP markers, CFB5887 and CFB5888, were present in many varieties. The presence of *im*-type alleles of three of the markers in the same varieties suggests that these are mutations of little agronomic consequence that reside together in a haplotype that has long been part of the global cotton germplasm, and are examples of identity by descent. Conversely, the lack of the *im* allele of the CFBid0001 marker in the diversity panel suggests that this mutation is relatively recent and is absent from cultivated germplasm. Indeed, this evidence is consistent with the history of the immature fiber mutation (Kohel *et al.* 1974).

### PPR genes affect mitochondrial gene expression

The PPR gene family consists of 450 genes in Arabidopsis and has been shown to play critical roles in all stages of organellar gene expression (Barkan and Small 2014). PPR proteins are specifically targeted to chloroplasts and mitochondria, where they bind single-stranded RNA (O’Toole *et al.* 2008; Barkan and Small 2014; Yin *et al.* 2013). Different PPR proteins associate with transcription and translation machineries and are involved in various aspects of organellar mRNA processing, including splicing, cleavage, and editing (Barkan and Small 2014). Since the crystal structure of the PPR domain bound to RNA has been solved, a “PPR-code” has been proposed based on the nucleotide specifying residues in each repeat (Yagi *et al.* 2013; Yin *et al.* 2013; Gully *et al.* 2015). Our analysis of transcripts in *im* mutant and wild-type parental NILs indicates that the 22-bp deletion should abolish the function of the PPR gene Gh_A03G0489. Of the reading frames that are present in the mutant transcript, one contains essentially only the transit peptide, while another contains most of the RNA-binding domain, but importantly lacks the transit peptide (Figure 5 and Figure S3). N-terminal transit peptides are required for import to chloroplasts and mitochondria, and can be specific to either or both (Emanuelsson *et al.* 2000). Analysis of Gh_A03G0489 indicates that it contains a mitochondria-specific transit peptide and 25 PPR repeats with a specific predicted RNA binding motif (Figure 6 and Table S3).

### Role of mitochondria in the development of cotton fiber properties

Our earlier work strongly implicated mitochondrial function in the development of the immature fiber mutant phenotype. We previously observed alteration of transcript levels of 12 genes involved in the cytochrome *c* oxidase respiration pathway, and activation of the alternative oxidase respiration machinery in mitochondria of the *im* mutant (Kim *et al.* 2013b). As the results of biochemical analysis in addition to the cotton microarray data, we suggested that deregulations of mitochondrial respiration in the *im* mutant fibers could cause energy deprivation that might reduce the degree of wall thickness of the *im* mutant fibers (Kim *et al.* 2013b). The verification of the significant upregulation (321-fold) of alternative oxidase (Gh_A12G2493/ Gh_D12G2621) by RNA-seq in the actively developing *im* mutant fibers at 28 DPA consistently indicated mitochondrial dysfunction in the *im* mutant fibers. Similarly, in *Arabidopsis*, disruption of the mitochondria-targeted PPR40 gene by T-DNA insertional mutagenesis interrupted the mitochondrial cytochrome pathway and activated the alternative pathway (Zsigmond *et al.* 2008). Our group has also identified altered mitochondrial gene expression in a different cotton fiber mutant, underscoring the importance of mitochondria in cotton fiber development and inviting future study (Thyssen *et al.* 2014b). Here, we have presented evidence of complete linkage of a frame-shifted PPR gene Gh_A03G0489 to the *im* allele that directly supports the association between mitochondrial dysfunction and mutant fiber phenotypes (Kim *et al.* 2013b). Further work is required to identify the specific RNA targets of the *im* candidate PPR gene Gh_A03G0489 in cotton mitochondria, and the direct functional consequence. Although mitochondrial dysfunction can clearly compromise fiber quality, it is not a foregone conclusion that wild-type mitochondrial function can be altered to improve fiber quality. However, researchers can already begin mining cotton genetic diversity for alternative alleles of PPR genes in the hope of fine-tuning maturity and fineness by affecting mitochondrial gene expression, to the benefit of cotton breeders, growers, and consumers.

## Supplementary Material

Supplemental Material
