# Supplementary material for: The Immature Fiber Mutant Phenotype of Cotton (Gossypium hirsutum) Is Linked to a 22-bp Frame-Shift Deletion in a Mitochondria Targeted Pentatricopeptide Repeat Gene
Source: G3 (Bethesda). 2016 Mar 29;6(6):1627–33. doi: 10.1534/g3.116.027649 (PMC4889659; doi:10.1534/g3.116.027649)
Supplement: Supplemental Material [file supp_6_6_1627__index.html]

The Immature Fiber Mutant Phenotype of Cotton (Gossypium hirsutum) Is Linked to a 22-bp Frame-Shift Deletion in a Mitochondria Targeted Pentatricopeptide Repeat Gene — Supplemental Material 

# The Immature Fiber Mutant Phenotype of Cotton (*Gossypium hirsutum*) Is Linked to a 22-bp Frame-Shift Deletion in a Mitochondria Targeted Pentatricopeptide Repeat Gene

## Supplemental Material for Thyssen *et al.*, 2016

**Files in this Data Supplement:**

- Figure S1 - Gene expression near the *im* locus in fiber cells by RT-qPCR. (.pdf, 367 KB)
- Figure S2 - Alignment of *im* and TM-1 alleles of PPR Gh\_ A03G0489. (.pdf, 295 KB)
- Figure S3 - Open Reading Frames in PPR Gh\_A030489 transcripts from *im* and TM-1 NILs. (.pdf, 283 KB)
- Table S1 - Diversity panel of 163 *Gossypium hirsutum* accessions genotyped for four markers near the *im* locus. (.pdf, 443 KB)
- Table S2 - Primer sequences. (.pdf, 192 KB)
- Table S3 - Domains of PPR Gh\_ A03G0489 and nucleotide binding site position-specific weight matrix. (.pdf, 344 KB)
