# Supplementary material for: The Immature Fiber Mutant Phenotype of Cotton (Gossypium hirsutum) Is Linked to a 22-bp Frame-Shift Deletion in a Mitochondria Targeted Pentatricopeptide Repeat Gene
Source: G3 (Bethesda). 2016 Mar 29;6(6):1627–33. doi: 10.1534/g3.116.027649 (PMC4889659; doi:10.1534/g3.116.027649)
Supplement: Supplemental Material [file supp_g3.116.027649_FigureS1.pdf]

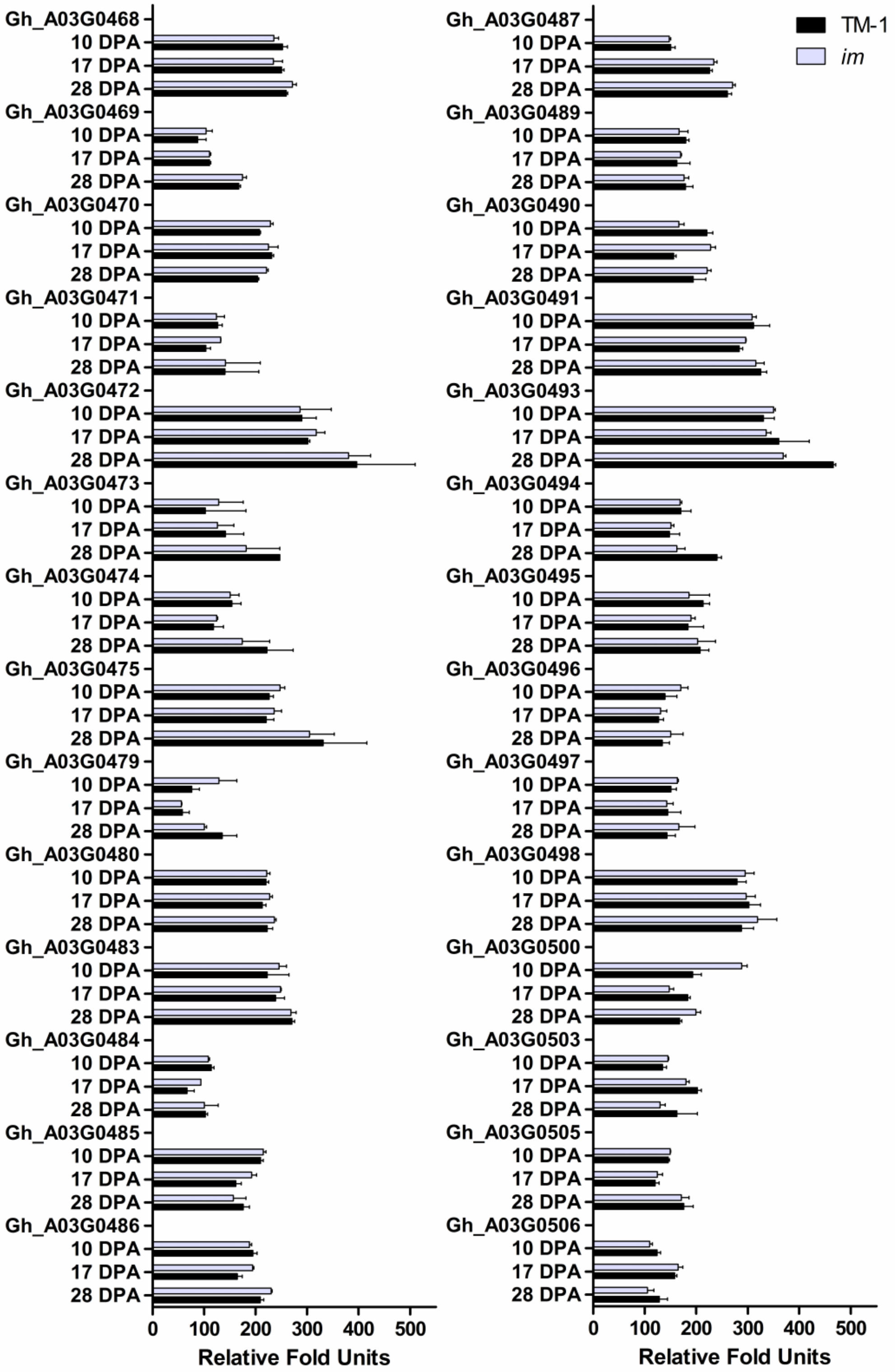
TM-1
im
Gh_A03G0468
Gh_A03G0469
Gh_A03G0470
Gh_A03G0471
Gh_A03G0472
Gh_A03G0473
Gh_A03G0474
Gh_A03G0475
Gh_A03G0479
Gh_A03G0480
Gh_A03G0483
Gh_A03G0484
Gh_A03G0485
Gh_A03G0486
Gh_A03G0487
Gh_A03G0489
Gh_A03G0490
Gh_A03G0491
Gh_A03G0493
Gh_A03G0494
Gh_A03G0495
Gh_A03G0496
Gh_A03G0497
Gh_A03G0498
Gh_A03G0500
Gh_A03G0503
Gh_A03G0505
Gh_A03G0506
10 DPA
17 DPA
28 DPA
0
100
200
300
400
500
Relative Fold Units

Figure S1 Gene expression near the *im* locus in fiber cells by RT-qPCR. Expression is presented for three developmental time points: 10, 17, and 28 days post anthesis (DPA).
