# Supplementary material for: The Immature Fiber Mutant Phenotype of Cotton (Gossypium hirsutum) Is Linked to a 22-bp Frame-Shift Deletion in a Mitochondria Targeted Pentatricopeptide Repeat Gene
Source: G3 (Bethesda). 2016 Mar 29;6(6):1627–33. doi: 10.1534/g3.116.027649 (PMC4889659; doi:10.1534/g3.116.027649)
Supplement: Supplemental Material [file supp_g3.116.027649_FigureS2.pdf]

```
                10        20        30        40        50        60
im      ATGCGTTTATCTTCAAAGCAAACGGTCTATTGTTTCACGAAATCAAGGCTCTCTCGTCTC
        ::::::::::::::::::::::::::::::::::::::::::::::::::::::::::::
TM-1    ATGCGTTTATCTTCAAAGCAAACGGTCTATTGTTTCACGAAATCAAGGCTCTCTCGTCTC
                10        20        30        40        50        60

                70        80        90       100       110       120
im      AAATCGGCTTCCCTTTGTACATCAGCTTCAAATGACGCCGAAGCCGTGGAAATCGCTTCT
        ::::::::::::::::::::::::::::::::::::::::::::::::::::::::::::
TM-1    AAATCGGCTTCCCTTTGTACATCAGCTTCAAATGACGCCGAAGCCGTGGAAATCGCTTCT
                70        80        90       100       110       120

               130       140       150       160       170       180
im      ATCCTCAGTAAAAATGACTGGAAACGACTTCTTGAAACATCATCAGAATTGACAAGCAAG
        ::::::::::::::::::::::::::::::::::::::::::::::::::::::::::::
TM-1    ATCCTCAGTAAAAATGACTGGAAACGACTTCTTGAAACATCATCAGAATTGACAAGCAAG
               130       140       150       160       170       180

               190       200       210       220       230       240
im      CTGAACCCAGAAATAGTTCACTCCGTTCTTCTCCAAAACTCGATTCATGATCCCGAACGC
        ::::::::::::::::::::::::::::::::::::::::::::::::::::::::::::
TM-1    CTGAACCCAGAAATAGTTCACTCCGTTCTTCTCCAAAACTCGATTCATGATCCCGAACGC
               190       200       210       220       230       240

               250       260       270       280       290       300
im      CTTTTCAGTTTCTTCAACTGGGCAAGTCAGCAATCCCCGAACCCTCAAAATTTGGATTCT
        ::::::::::::::::::::::::::::::::::::::::::::::::::::::::::::
TM-1    CTTTTCAGTTTCTTCAACTGGGCAAGTCAGCAATCCCCGAACCCTCAAAATTTGGATTCT
               250       260       270       280       290       300

               310       320       330       340       350       360
im      TTTTCTTTTCTAGCAGTTACTTTATGTAATTCGAAGATGTTTCGTAAAGCCACTATTGTT
        ::::::::::::::::::::::::::::::::::::::::::::::::::::::::::::
TM-1    TTTTCTTTTCTAGCAGTTACTTTATGTAATTCGAAGATGTTTCGTAAAGCCACTATTGTT
               310       320       330       340       350       360

               370       380       390       400       410       420
im      TTAGACAGCATGGTTAGGACTAGAAGGCCCGTTCAGGCCATTTTGGGTTCAATTATTAGG
        ::::::::::::::::::::::::::::::::::::::::::::::::::::::::::::
TM-1    TTAGACAGCATGGTTAGGACTAGAAGGCCCGTTCAGGCCATTTTGGGTTCAATTATTAGG
               370       380       390       400       410       420

               430       440       450       460       470       480
im      TGCTGGAAGGGCTATGAAGGGAATTATGTCGGGGTTTTCGATATTTTGATTGGTTGTTAT
        ::::::::::::::::::::::::::::::::::::::::::::::::::::::::::::
TM-1    TGCTGGAAGGGCTATGAAGGGAATTATGTCGGGGTTTTCGATATTTTGATTGGTTGTTAT
               430       440       450       460       470       480

               490       500       510       520       530       540
im      AAGAAAATGGGTTCTTGGAACGATGCTGTTTATGTTTTTTTGGGTGCTAAAGAAGTTGGG
        ::::::::::::::::::::::::::::::::::::::::::::::::::::::::::::
TM-1    AAGAAAATGGGTTCTTGGAACGATGCTGTTTATGTTTTTTTGGGTGCTAAAGAAGTTGGG
               490       500       510       520       530       540

                                 550       560       570
```

```
im      GTTTTGCCT----------------------TTTTAGGTGATTTGCTTAAGGTAAATAAG
        :::::::::                      :::::::::::::::::::::::::::::
TM-1    GTTTTGCCTGGTTTGGCTTGTTGTAATAACTTTTTAGGTGATTTGCTTAAGGTAAATAAG
                550       560       570       580       590       600

      580       590       600       610       620       630
im      TTGGATTTGTTTTGGAAGGTTTTCAATGGGATGATAGATGCTAAATTGGTGCCTGATGTT
        ::::::::::::::::::::::::::::::::::::::::::::::::::::::::::::
TM-1    TTGGATTTGTTTTGGAAGGTTTTCAATGGGATGATAGATGCTAAATTGGTGCCTGATGTT
                610       620       630       640       650       660

      640       650       660       670       680       690
im      TATACTTTTACCAATGTGATTAATGCTCATTGTCGAGTAGGGGATATTGAGAAGGCAAAA
        ::::::::::::::::::::::::::::::::::::::::::::::::::::::::::::
TM-1    TATACTTTTACCAATGTGATTAATGCTCATTGTCGAGTAGGGGATATTGAGAAGGCAAAA
                670       680       690       700       710       720

      700       710       720       730       740       750
im      AGGGTGTTTATGGATATGGAGGAGAAGGGATGTAGCCCTGGTTTGGTTACTTATAATGTT
        ::::::::::::::::::::::::::::::::::::::::::::::::::::::::::::
TM-1    AGGGTGTTTATGGATATGGAGGAGAAGGGATGTAGCCCTGGTTTGGTTACTTATAATGTT
                730       740       750       760       770       780

      760       770       780       790       800       810
im      ATGATTGGTGGACTGTGTAAAGCTGGTGCTGTTGATGAAGCATTGAAGTTGAAAAACTCC
        ::::::::::::::::::::::::::::::::::::::::::::::::::::::::::::
TM-1    ATGATTGGTGGACTGTGTAAAGCTGGTGCTGTTGATGAAGCATTGAAGTTGAAAAACTCC
                790       800       810       820       830       840

      820       830       840       850       860       870
im      ATGAGTCGGAAGGGTTTCAACCCTGATGCATATACTTACAATACGGTTATTAATGGGTTT
        ::::::::::::::::::::::::::::::::::::::::::::::::::::::::::::
TM-1    ATGAGTCGGAAGGGTTTCAACCCTGATGCATATACTTACAATACGGTTATTAATGGGTTT
                850       860       870       880       890       900

      880       890       900       910       920       930
im      TGTAGGCATAACAGATTTCAGGAAGCAAAATTGATGATGGAGGAAATGCGTTGTGCAGGC
        ::::::::::::::::::::::::::::::::::::::::::::::::::::::::::::
TM-1    TGTAGGCATAACAGATTTCAGGAAGCAAAATTGATGATGGAGGAAATGCGTTGTGCAGGC
                910       920       930       940       950       960

      940       950       960       970       980       990
im      TTAAATCCTAACCATTTTGCTTACACAGCTTTGATTGATGGGTTCATTAAACGGGGGAGC
        ::::::::::::::::::::::::::::::::::::::::::::::::::::::::::::
TM-1    TTAAATCCTAACCATTTTGCTTACACAGCTTTGATTGATGGGTTCATTAAACGGGGGAGC
                970       980       990      1000      1010      1020

     1000      1010      1020      1030      1040      1050
im      ATGGCCGAGGCTTTCCAAGTCAAGGATGAGATGGTTGCTTGTGGAATTAAACCGAATGTG
        ::::::::::::::::::::::::::::::::::::::::::::::::::::::::::::
TM-1    ATGGCCGAGGCTTTCCAAGTCAAGGATGAGATGGTTGCTTGTGGAATTAAACCGAATGTG
               1030      1040      1050      1060      1070      1080

     1060      1070      1080      1090      1100      1110
im      TTCACGTATAATGTGCTTATTGGTGGGGCTTCTAAGGCTGGTGACTTGGAGAAAGGAAAA
        ::::::::::::::::::::::::::::::::::::::::::::::::::::::::::::
TM-1    TTCACGTATAATGTGCTTATTGGTGGGGCTTCTAAGGCTGGTGACTTGGAGAAAGGAAAA
```

```
           1090      1100      1110      1120      1130      1140

     1120      1130      1140      1150      1160      1170
im     GCTCTGTTCGATGAGATGGTTTTGATTGGCATCGGACCTGATGCACAAACATTTAGTCTT
       ::::::::::::::::::::::::::::::::::::::::::::::::::::::::::::
TM-1   GCTCTGTTCGATGAGATGGTTTTGATTGGCATCGGACCTGATGCACAAACATTTAGTCTT
           1150      1160      1170      1180      1190      1200

     1180      1190      1200      1210      1220      1230
im     ATGATCCAGAGTTATTGTCGACAAGGGAATTTTCTCAAGGCCCATGAATTGTTTGAAGAG
       ::::::::::::::::::::::::::::::::::::::::::::::::::::::::::::
TM-1   ATGATCCAGAGTTATTGTCGACAAGGGAATTTTCTCAAGGCCCATGAATTGTTTGAAGAG
           1210      1220      1230      1240      1250      1260

     1240      1250      1260      1270      1280      1290
im     ATGAAGGAACACAACTTGACACCCACGATGTACACTTATAATGGTATAATTAGTGGATTA
       ::::::::::::::::::::::::::::::::::::::::::::::::::::::::::::
TM-1   ATGAAGGAACACAACTTGACACCCACGATGTACACTTATAATGGTATAATTAGTGGATTA
           1270      1280      1290      1300      1310      1320

     1300      1310      1320      1330      1340      1350
im     TGTCACTGTGGAGATCTTGGTCGAGCTAATTATGTTTTTGAGGCAATGATTAAGGTAGGG
       ::::::::::::::::::::::::::::::::::::::::::::::::::::::::::::
TM-1   TGTCACTGTGGAGATCTTGGTCGAGCTAATTATGTTTTTGAGGCAATGATTAAGGTAGGG
           1330      1340      1350      1360      1370      1380

     1360      1370      1380      1390      1400      1410
im     TTGAAGCCCAACGTTGTTATGTATACAAATCTGATAAAAGGTCATATTCAGAAAAGTAGA
       ::::::::::::::::::::::::::::::::::::::::::::::::::::::::::::
TM-1   TTGAAGCCCAACGTTGTTATGTATACAAATCTGATAAAAGGTCATATTCAGAAAAGTAGA
           1390      1400      1410      1420      1430      1440

     1420      1430      1440      1450      1460      1470
im     TTCAAGGAAGCAAGAAAGATTCTCAACAGAATGATGGAGAATGGACAAATGGCTGATATC
       ::::::::::::::::::::::::::::::::::::::::::::::::::::::::::::
TM-1   TTCAAGGAAGCAAGAAAGATTCTCAACAGAATGATGGAGAATGGACAAATGGCTGATATC
           1450      1460      1470      1480      1490      1500

     1480      1490      1500      1510      1520      1530
im     TTTTGTTGCAATACTCTTTTAAGTGGCTTATGTAAGTCCCAAATGATGGACGAAGCAAGG
       ::::::::::::::::::::::::::::::::::::::::::::::::::::::::::::
TM-1   TTTTGTTGCAATACTCTTTTAAGTGGCTTATGTAAGTCCCAAATGATGGACGAAGCAAGG
           1510      1520      1530      1540      1550      1560

     1540      1550      1560      1570      1580      1590
im     AGCTTCTTAGTCGAAATGGTTGACAGGGGGTTGAAGCCTAATGAAATTACCTATGGGACT
       ::::::::::::::::::::::::::::::::::::::::::::::::::::::::::::
TM-1   AGCTTCTTAGTCGAAATGGTTGACAGGGGGTTGAAGCCTAATGAAATTACCTATGGGACT
           1570      1580      1590      1600      1610      1620

     1600      1610      1620      1630      1640      1650
im     TTAATCCATGGATATGCTAAGGCCGGGGAAATGGAGGCAGTAGAAAGGTGTTTCAGAGAG
       ::::::::::::::::::::::::::::::::::::::::::::::::::::::::::::
TM-1   TTAATCCATGGATATGCTAAGGCCGGGGAAATGGAGGCAGTAGAAAGGTGTTTCAGAGAG
           1630      1640      1650      1660      1670      1680

     1660      1670      1680      1690      1700      1710
```

```
im      ATGCGTAGTTATGGTATAGCTCCAAATAATGCTATCTATACTATATTGATTAATAGCCTT
        ::::::::::::::::::::::::::::::::::::::::::::::::::::::::::::
TM-1    ATGCGTAGTTATGGTATAGCTCCAAATAATGCTATCTATACTATATTGATTAATAGCCTT
              1690      1700      1710      1720      1730      1740

    1720      1730      1740      1750      1760      1770
im      TGCAAAGTGGGAAATGTAACCGAGGCTCTGTCGACATTTAGGCACATGTCTGAAAAAGGT
        ::::::::::::::::::::::::::::::::::::::::::::::::::::::::::::
TM-1    TGCAAAGTGGGAAATGTAACCGAGGCTCTGTCGACATTTAGGCACATGTCTGAAAAAGGT
              1750      1760      1770      1780      1790      1800

    1780      1790      1800      1810      1820      1830
im      GTGCTCCCAGACATAAAGACTTACACTGCACTCATCCACGGCCTTGCAGCAAATGGTAGA
        ::::::::::::::::::::::::::::::::::::::::::::::::::::::::::::
TM-1    GTGCTCCCAGACATAAAGACTTACACTGCACTCATCCACGGCCTTGCAGCAAATGGTAGA
              1810      1820      1830      1840      1850      1860

    1840      1850      1860      1870      1880      1890
im      ATTAATGATGCAATGCAACTGTTCTCTCAACTTGATGAAAAGGGTATAGTGCCTGATGTA
        ::::::::::::::::::::::::::::::::::::::::::::::::::::::::::::
TM-1    ATTAATGATGCAATGCAACTGTTCTCTCAACTTGATGAAAAGGGTATAGTGCCTGATGTA
              1870      1880      1890      1900      1910      1920

    1900      1910      1920      1930      1940      1950
im      TTCACTTATACTTCTCTCATTTCTGTCTTTTCTAAGCTTGGTGATATGGAGGCTGCTCTC
        ::::::::::::::::::::::::::::::::::::::::::::::::::::::::::::
TM-1    TTCACTTATACTTCTCTCATTTCTGTCTTTTCTAAGCTTGGTGATATGGAGGCTGCTCTC
              1930      1940      1950      1960      1970      1980

    1960      1970      1980      1990      2000      2010
im      AACCTTTATGATAAGATGTGTCAAAAGGGCATTGCTCCTAATATTGTTACATATAATGCA
        ::::::::::::::::::::::::::::::::::::::::::::::::::::::::::::
TM-1    AACCTTTATGATAAGATGTGTCAAAAGGGCATTGCTCCTAATATTGTTACATATAATGCA
              1990      2000      2010      2020      2030      2040

    2020      2030      2040      2050      2060      2070
im      ATGATTGATGGTCTGTGTAAGTTAGGTGACACCGAGAAAGCTAGGAGGGTGTTTAATGAA
        ::::::::::::::::::::::::::::::::::::::::::::::::::::::::::::
TM-1    ATGATTGATGGTCTGTGTAAGTTAGGTGACACCGAGAAAGCTAGGAGGGTGTTTAATGAA
              2050      2060      2070      2080      2090      2100

    2080      2090      2100      2110      2120      2130
im      ATCGCACAGAGAGGCTTGGCACCTAATGTCAAGAGTTATTCTATAATTATAGATGGATAT
        ::::::::::::::::::::::::::::::::::::::::::::::::::::::::::::
TM-1    ATCGCACAGAGAGGCTTGGCACCTAATGTCAAGAGTTATTCTATAATTATAGATGGATAT
              2110      2120      2130      2140      2150      2160

    2140      2150      2160      2170      2180      2190
im      TGCAAATCTGGAAATTTAACTGAGGCATTTCAGTTACTGGATAAAATGCCCTCAAAGGGT
        ::::::::::::::::::::::::::::::::::::::::::::::::::::::::::::
TM-1    TGCAAATCTGGAAATTTAACTGAGGCATTTCAGTTACTGGATAAAATGCCCTCAAAGGGT
              2170      2180      2190      2200      2210      2220

    2200      2210      2220      2230      2240      2250
im      GTTCCACCTGATAGTTTTACTTATTGTGCCCTTATTGACGGATGCTGTAAAGGAGGAGAA
        ::::::::::::::::::::::::::::::::::::::::::::::::::::::::::::
TM-1    GTTCCACCTGATAGTTTTACTTATTGTGCCCTTATTGACGGATGCTGTAAAGGAGGAGAA
```

```
          2230      2240      2250      2260      2270      2280

    2260      2270      2280      2290      2300      2310
im      TTGGACAAGGCGCTCTCTTTGTTTTCTGAAATGTTGCAGAAGGGTTTTGATCCCACCTCT
        ::::::::::::::::::::::::::::::::::::::::::::::::::::::::::::
TM-1    TTGGACAAGGCGCTCTCTTTGTTTTCTGAAATGTTGCAGAAGGGTTTTGATCCCACCTCT
              2290      2300      2310      2320      2330      2340

    2320      2330      2340      2350      2360      2370
im      TCTTTCAATGCATTGATCAATGGGTTATGCAAGACGGGGAAACCAAATGAAGCTATGAGG
        ::::::::::::::::::::::::::::::::::::::::::::::::::::::::::::
TM-1    TCTTTCAATGCATTGATCAATGGGTTATGCAAGACGGGGAAACCAAATGAAGCTATGAGG
              2350      2360      2370      2380      2390      2400

    2380      2390      2400      2410      2420      2430
im      CTGTTAGAAGACATGGCCAGTAACTGTATTACACCAAACCATAGAACCTATACAATCCTA
        ::::::::::::::::::::::::::::::::::::::::::::::::::::::::::::
TM-1    CTGTTAGAAGACATGGCCAGTAACTGTATTACACCAAACCATAGAACCTATACAATCCTA
              2410      2420      2430      2440      2450      2460

    2440      2450      2460      2470      2480      2490
im      ATTGATTATCACAGCAAGGCAGGAAGGATGGAAGAAGCCGAAATTCTCTTTTTGGAGATG
        ::::::::::::::::::::::::::::::::::::::::::::::::::::::::::::
TM-1    ATTGATTATCACAGCAAGGCAGGAAGGATGGAAGAAGCCGAAATTCTCTTTTTGGAGATG
              2470      2480      2490      2500      2510      2520

    2500      2510      2520      2530      2540      2550
im      CAAAGGAGGAATCTGGTTCCGAATGTTGTAACTTACAACTTGCTTTTACATGGTTACTAC
        ::::::::::::::::::::::::::::::::::::::::::::::::::::::::::::
TM-1    CAAAGGAGGAATCTGGTTCCGAATGTTGTAACTTACAACTTGCTTTTACATGGTTACTAC
              2530      2540      2550      2560      2570      2580

    2560      2570      2580      2590      2600      2610
im      AGATTAGGGAGGAAAGCAGAGATGTTTGATCTTTTCGAGTCCATGGCTGCAAAAGGGGTT
        ::::::::::::::::::::::::::::::::::::::::::::::::::::::::::::
TM-1    AGATTAGGGAGGAAAGCAGAGATGTTTGATCTTTTCGAGTCCATGGCTGCAAAAGGGGTT
              2590      2600      2610      2620      2630      2640

    2620      2630      2640      2650      2660      2670
im      GAACCTGATGAAATTATTTATCGTCTTATTGCTAATGCTTACTTGGAAGAAAATAGTTTG
        ::::::::::::::::::::::::::::::::::::::::::::::::::::::::::::
TM-1    GAACCTGATGAAATTATTTATCGTCTTATTGCTAATGCTTACTTGGAAGAAAATAGTTTG
              2650      2660      2670      2680      2690      2700

    2680      2690      2700      2710      2720      2730
im      ATTGGAATGTTGAAGCTGTTGGATGAAATTCTAGTGAAGGATGTTGTGTTCGATAAAAAT
        ::::::::::::::::::::::::::::::::::::::::::::::::::::::::::::
TM-1    ATTGGAATGTTGAAGCTGTTGGATGAAATTCTAGTGAAGGATGTTGTGTTCGATAAAAAT
              2710      2720      2730      2740      2750      2760

    2740      2750      2760      2770      2780      2790
im      CCCACTTTTCTGCTGCTGGACGCTGTATGCAAAAGAGAGGAGTTTTCGGAAGTTCCAAAG
        ::::::::::::::::::::::::::::::::::::::::::::::::::::::::::::
TM-1    CCCACTTTTCTGCTGCTGGACGCTGTATGCAAAAGAGAGGAGTTTTCGGAAGTTCCAAAG
              2770      2780      2790      2800      2810      2820

    2800      2810      2820      2830      2840      2850
```

```
im     TCACTTGAAGAAATGGCAGAACAAGGACTTAAGCTCAGTCCAATTACATGTCACAAGCTT
       ::::::::::::::::::::::::::::::::::::::::::::::::::::::::::::
TM-1   TCACTTGAAGAAATGGCAGAACAAGGACTTAAGCTCAGTCCAATTACATGTCACAAGCTT
             2830      2840      2850      2860      2870      2880

     2860      2870      2880      2890      2900      2910
im     GTTCATGGTTTTCATGATAAGGGAAATCCAGAGAAAGCAGAATGGATTCTTGAGAGCTTG
       ::::::::::::::::::::::::::::::::::::::::::::::::::::::::::::
TM-1   GTTCATGGTTTTCATGATAAGGGAAATCCAGAGAAAGCAGAATGGATTCTTGAGAGCTTG
             2890      2900      2910      2920      2930      2940

     2920      2930      2940      2950      2960      2970
im     GTCCGGTTCGGATGGATTCCAAACACCACCACTGTGAATAGCATTATTGATAAAGAAAAT
       ::::::::::::::::::::::::::::::::::::::::::::::::::::::::::::
TM-1   GTCCGGTTCGGATGGATTCCAAACACCACCACTGTGAATAGCATTATTGATAAAGAAAAT
             2950      2960      2970      2980      2990      3000

     2980      2990      3000      3010      3020      3030
im     GATGTTGCAAACTTGGAGAGCCCTAACAATTCTCCCAAGCAAGCAACGTGCGGAGTTGCT
       ::::::::::::::::::::::::::::::::::::::::::::::::::::::::::::
TM-1   GATGTTGCAAACTTGGAGAGCCCTAACAATTCTCCCAAGCAAGCAACGTGCGGAGTTGCT
             3010      3020      3030      3040      3050      3060

     3040      3050
im     TGTCAAGTGTAA
       ::::::::::::
TM-1   TGTCAAGTGTAA
             3070
```

Figure S2 Alignment of *im* and TM-1 alleles of PPR Gh_ A03G0489. Primer locations for CFBid0001 are highlighted.
