# Supplementary material for: The Immature Fiber Mutant Phenotype of Cotton (Gossypium hirsutum) Is Linked to a 22-bp Frame-Shift Deletion in a Mitochondria Targeted Pentatricopeptide Repeat Gene
Source: G3 (Bethesda). 2016 Mar 29;6(6):1627–33. doi: 10.1534/g3.116.027649 (PMC4889659; doi:10.1534/g3.116.027649)
Supplement: Supplemental Material [file supp_g3.116.027649_FigureS3.pdf]

im-Frame 1

MRLSSKQTVYCFTKSRLSRLKSASLCTSASNDAEAVEIASILSKNDWKRLLETSSELTSK
LNPEIVHSVLLQNSIHDPERLFSFFNWASQQSPNPQNLDSFSFLAVTLCNSKMFRKATIV
LDSMVRTRRPVQAILGSIIRCWKGYEGNYVGVFDILIGCYKKMGSWNDAVYVFLGAKEVG
VLPF-VICLR-ISWICFGRFSMG--MLNWCLMFILLPM-LMLIVE-GILRRQKGCLWIWR
RRDVALVWLLIML-LVDCVKLVLLMKH-S-KTP-VGRVSTLMHILTIRLLMGFVGITDFR
KQN--WRKCVVQA-ILTILLTQL-LMGSLNGGAWPRLSKSRMRWLLVELNRMCSRIMCLL
VGLLRLVTWRKEKLCSMRWF-LASDLMHKHLVL-SRVIVDKGIFSRPMNCLKR-RNTT-H
PRCTLIMV-LVDYVTVEILVELIMFLRQ-LR-G-SPTLLCIQI--KVIFRKVDSRKQERF
STE-WRMDKWLISFVAILF-VAYVSPK-WTKQGAS-SKWLTGG-SLMKLPMGL-SMDMLR
PGKWRQ-KGVSERCVVMV-LQIMLSILY-LIAFAKWEM-PRLCRHLGTCLKKVCSQT-RL
TLHSSTALQQMVELMMQCNCSLNLMKRV-CLMYSLILLSFLSFLSLVIWRLLSTFMIRCV
KRALLLILLHIMQ-LMVCVS-VTPRKLGGCLMKSHREAWHLMSRVIL-L-MDIANLEI-L
RHFSYWIKCPQRVFHLIVLLIVPLLTDAVKEENWTRRSLCFLKCCRRVLIPPLLSMH-SM
GYARRGNQMKL-GC-KTWPVTVLHQTIEPIQS-LIITARQEGWKKPKFSFWRCKGGIWFR
ML-LTTCFYMVTTD-GGKQRCLIFSSPWLQKGLNLMKLFIVLLLMLTWKKIV-LEC-SCW
MKF--RMLCSIKIPLFCCWTLYAKERSFRKFQSHLKKWQNKDLSSVQLHVTSLFMVFMIR
EIQRKQNGFLRAWSGSDGFQTPPL-IALLIKKMMLQTWRALTILPSKQRAELLVKC

im-Frame 3

AFIFKANGLLFHEIKALSSQIGFPLYISFK-RRSRGNRFYPQ-K-LETTS-NIIRIDKQA
EPRNSSLRSSPKLDS-SRTPFQFLQLGKSAIPEPSKFGFFFFSSSYFM-FEDVS-SHYCF
RQHG-D-KARSGHFGFNY-VLEGL-RELCRGFRYFDWLL-ENGFLERCCLCFFGC-RSWG
FAFLGDLLKVNKLDLFWKVFNGMIDAKLVPDVYTFTNVINAHCRVGDIEKAKRVFMDMEE
KGCSPGLVTYNVMIGGLCKAGAVDEALKLKNSMSRKGFNPDAYTYNTVINGFCRHNRFQE
AKLMMEEMRCAGLNPNHFAYTALIDGFIKRGSMAEAFQVKDEMVACGIKPNVFTYNVLIG
GASKAGDLEKGKALFDEMVLIGIGPDAQTFSLMIQSYCRQGNFLKAHELFEEMKEHNLTP
TMYTYNGIISGLCHCGDLGRANYVFEAMIKVGLKPNVVMYTNLIKGHIQKSRFKEARKIL
NRMMENGQMADIFCCNTLLSGLCKSQMMDEARSFLVEMVDRGLKPNEITYGTLIHGYAKA
GEMEAVERCFREMRSYGIAPNNAIYTILINSLCKVGNVTEALSTFRHMSEKGVLPDIKTY
TALIHGLAANGRINDAMQLFSQLDEKGIVPDVFTYTSLISVFSKLGDMEAALNLYDKMCQ
KGIAPNIVTYNAMIDGLCKLGDTEKARRVFNEIAQRGLAPNVKSYSIIIDGYCKSGNLTE
AFQLLDKMPSKGVPPDSFTYCALIDGCCKGGELDKALSLFSEMLQKGFDPTSSFNALING
LCKTGKPNEAMRLLEDMASNCITPNHRTYTILIDYHSKAGRMEEAEILFLEMQRRNLVPN
VVTYNLLLHGYYRLGRKAEMFDLFESMAAKGVEPDEIIYRLIANAYLEENSLIGMLKLLD
EILVKDVVFDKNPTFLLLDAVCKREEFSEVPKSLEEMAEQGLKLSPITCHKLVHGFHDKG
NPEKAEWILESLVRFGWIPNTTTVNSIIDKENDVANLESPNNSPKQATCGVACQV-

TM-1-Frame 1

MRLSSKQTVYCFTKSRLSRLKSASLCTSASNDAEAVEIASILSKNDWKRLLETSSELTSK
LNPEIVHSVLLQNSIHDPERLFSFFNWASQQSPNPQNLDSFSFLAVTLCNSKMFRKATIV
LDSMVRTRRPVQAILGSIIRCWKGYEGNYVGVFDILIGCYKKMGSWNDAVYVFLGAKEVG
VLPGLACCNNFLGDLLKVNKLDLFWKVFNGMIDAKLVPDVYTFTNVINAHCRVGDIEKAK
RVFMDMEEKGCSPGLVTYNVMIGGLCKAGAVDEALKLKNSMSRKGFNPDAYTYNTVINGF
CRHNRFQEAKLMMEEMRCAGLNPNHFAYTALIDGFIKRGSMAEAFQVKDEMVACGIKPNV
FTYNVLIGGASKAGDLEKGKALFDEMVLIGIGPDAQTFSLMIQSYCRQGNFLKAHELFEE
MKEHNLTPTMYTYNGIISGLCHCGDLGRANYVFEAMIKVGLKPNVVMYTNLIKGHIQKSR
FKEARKILNRMMENGQMADIFCCNTLLSGLCKSQMMDEARSFLVEMVDRGLKPNEITYGT
LIHGYAKAGEMEAVERCFREMRSYGIAPNNAIYTILINSLCKVGNVTEALSTFRHMSEKG
VLPDIKTYTALIHGLAANGRINDAMQLFSQLDEKGIVPDVFTYTSLISVFSKLGDMEAAL
NLYDKMCQKGIAPNIVTYNAMIDGLCKLGDTEKARRVFNEIAQRGLAPNVKSYSIIIDGY
CKSGNLTEAFQLLDKMPSKGVPPDSFTYCALIDGCCKGGELDKALSLFSEMLQKGFDPTS

SFNALINGLCKTGKPNEAMRLLEDMASNCITPNHRTYTILIDYHSKAGRMEEAEILFLEM
QRRNLVPNVVTYNLLLHGYYRLGRKAEMFDLFESMAAKGVEPDEIIYRLIANAYLEENSL
IGMLKLLDEILVKDVVFDKNPTFLLLDAVCKREEFSEVPKSLEEMAEQGLKLSPITCHKL
VHGFHDKGNPEKAEWILESLVRFGWIPNTTTVNSIIDKENDVANLESPNNSPKQATCGVA
CQV-

Figure S3 Open Reading Frames in PPR Gh_A030489 transcripts from *im* and TM-1 NILs
