# Supplementary material for: The Immature Fiber Mutant Phenotype of Cotton (Gossypium hirsutum) Is Linked to a 22-bp Frame-Shift Deletion in a Mitochondria Targeted Pentatricopeptide Repeat Gene
Source: G3 (Bethesda). 2016 Mar 29;6(6):1627–33. doi: 10.1534/g3.116.027649 (PMC4889659; doi:10.1534/g3.116.027649)
Supplement: Supplemental Material [file supp_g3.116.027649_TableS1.pdf]

Table S1 Diversity panel of 163 *Gossypium hirsutum* accessions genotyped for four markers near the *im* locus.

Variety Name	CFB 5887	CFB 5888	CFBid 0001	CFBid 0002	SA#	PI#	Country of Origin
1OE	A	A	A	A	SA-1441	PI 361150	Greece
320F	B	B	A	B	SA-1167	PI 529233	India
4S-180	H	H	A	H	SA-1442	PI 361151	Greece
A-618	A	A	A	A	SA-1297	PI 408996	Mozambique
A-637-33	A	H	A	H	SA-1300	PI 408999	Mozambique
Acala 1517 (New Mexico)	A	A	A	A	SA-0239	PI 528607	USA
Acala Maxxa	A	H	A	H			USA
Acala Nemex	B	B	A	B	SA-2289	PI 590568	USA
Acala Royale	B	B	A	A	SA-3778	PI 542049	USA
AK Djura 182	H	H	A	A	SA-1202	364267	Uzbekistan
ALA 70-236	A	A	A	A	SA-1283	PI 402499	Swaziland
Albar 627	H	H	A	H	SA-1440	PI 529494	Zambia
Aleppo I	H	H	A	H	SA-1395	529450	Syria
Allen 333	A	A	A	A	SA-1262	529318	Togo
ALTIMA	H	H	A	B			USA
Arkot 8606	H	H	A	A	SA-3112	PI 628634	USA
ATLAS	B	B	A	B			USA
Auburn 56	A	A	A	A	SA-1149	529215	USA
Auburn 634 RNR	A	A	A	A	SA-2413	607237	USA
AUSTIN	H	H	A	A			USA
Beli Izvor	H	H	A	H	SA-1724	PI 532789	Bulgaria
Big Boll Triumph UA 8-20 (P1)	A	A	A	A	SA-0148	606883	Trinidad & Tobago
BLIGHTMASTER	A	A	A	A			USA
BPA 68 CB 4030	H	H	A	B	SA-1243	PI 365538	Uganda
Bulgaria P73	A	H	A	H	SA-1407	529462	Bulgaria
CABD3SHP3S-1-90	A	A	A	A	SA-2388	PI 603007	USA
CAHUGLBBCS-1-88	A	A	A	A	SA-2386	PI 603005	USA
Cambodia #4	A	A	A	A	SA-0501	528805	Cambodia
CB 4011 Dehkanin	B	B	A	B	SA-1204	PI 364269	Uzbekistan
CD3HCHULBH-1-88	A	A	A	A	SA-2382	PI 603001	USA
Central (Sel	H	H	A	A	SA-1210	PI 529275	Venezuela

Variety Name	CFB 5887	CFB 5888	CFBid 0001	CFBid 0002	SA#	PI#	Country of Origin
Coker 100)							
CHACO 510 INTA	A	A	A	A	SA-1759	PI 606817	Argentina
Christidis	A	A	A	A	SA-1166	PI 529232	Greece
Ciano Cocorim 92	A	A	A	A	SA-2206	PI 570666	Mexico
Cleveland W.R. Wannamaker's	H	H	A	H	SA-0296	528645	USA
Coker 100 WILT	A	A	A	A	SA-0451	528761	USA
COKER 100A	H	H	A	B			USA
COKER 124	B	B	A	B			USA
Coker 312	A	A	A	A	SA-1213	PI 529278	USA
COKER 315	A	A	A	A			USA
Coker's Clevewilt #3	A	H	A	A	SA-0253	528617	USA
Colombia 1 Gossica N21 VIR-7263	H	H	A	B	SA-3475	n/a	Colombia
Columbia	A	A	A	A	SA-0429	528743	Columbia
Cook 912 Pope	B	B	A	B	SA-0053	528466	USA
Cristina	A	A	A	A	SA-1757	606815	Spain
Dekalb 220	A	A	A	A	SA-1156	529222	USA
Del Cerro	A	A	A	A	SA-1302	529358	Brazil
Delcot 277	A	A	A	A	SA-1193	529258	USA
Delfos 9169	A	A	A	A	SA-0309	528655	USA
Deltapine Smoothleaf	H	H	A	H	SA-1153	PI 529219	USA
Deltapine 10-1	B	B	A	B	SA-0369	PI 528699	USA
Deltapine 12	B	B	A	B	SA-0459	PI 528768	USA
Deltapine 15	H	H	A	B			USA
Deltapine 20	B	B	A	B	SA-1513	PI 529567	USA
Deltapine 25	H	H	A	H	SA-1215	PI 529280	USA
Deltapine 393	H	B	A	B			USA
Deltapine 491	H	H	A	H	SA-3139	PI 618609	USA
Deltapine 50	B	B	A	B	SA-1512	PI 529566	USA
Deltapine 5409	A	A	A	A			USA
Deltapine 5415	H	H	A	B			USA
Deltapine 5690	A	A	A	A			USA
Deltapine 6	B	A	A	A	SA-0870	PI 528969	USA
Deltapine 66	A	A	A	A	SA-1511	PI 529565	USA
Deltapine 80	B	B	A	B	SA-2297	PI 607179	USA

Variety Name	CFB 5887	CFB 5888	CFBid 0001	CFBid 0002	SA#	PI#	Country of Origin
Deltapine 826	B	B	A	B	SA-2294	PI 607176	USA
Deltapine 90	A	A	A	A			USA
DES 119	A	A	A	A			USA
DES 716	B	B	A	B	SA-0998	PI 529067	USA
Dixie King	A	A	A	A	SA-0951	529021	USA
DIXIE KING II	A	A	A	B			USA
Dixie Triumph	B	B	A	B	SA-0852	528956	USA
Dixie Triumph Wannamaker's	B	B	A	B	SA-0294	528643	USA
Dunn 219	B	B	A	B	SA-2377	PI 600744	USA
Dunn 325	H	H	A	B	SA-3209	601199	USA
Empire W.R. 61	A	A	A	A	SA-1158	529224	USA
Felistana UA-7-18	A	A	A	A	SA-0186	606910	Trinidad & Tobago
Fibermax 832	B	B	A	B			USA
Fibermax 958	H	H	A	A			Australia
Fibermax 966	H	H	A	A			Australia
Fibermax 989	A	A	A	A			Australia
GA 230	A	A	A	A			USA
GA 4303	A	A	A	A			USA
GA 98028	H	H	A	H	SA-3182	PI 633796	USA
GA KING	A	A	A	A			USA
Gregg	A	A	A	A	SA-1025	529094	USA
Gringo	A	A	A	A	SA-1760	PI 606818	Argentina
Guazuncho II	H	H	A	A	SA-1761	PI 606819	Argentina
HS26	H	H	A	H			USA
IAC 17	A	A	A	A	SA-1348	PI 438980	Brazil
IAC 18	A	A	A	A	SA-1349	PI 438981	Brazil
im	B	B	B	B			
LA 17	A	H	A	H			USA
Lambright 2020A	B	B	A	B	SA-2895	592517	USA
Lankart	H	H	A	A	SA-3185	PI 601147	USA
LANKART 57	B	B	A	B			USA
Lightning Express	A	A	A	A	SA-0883	528978	USA
Limpopo	A	A	A	A	SA-2332	607199	S. Africa
Lisina 11 CB 4021	H	A	A	A	SA-1234	PI 365529	Pakistan
Lockett BXL	H	H	A	A	SA-1533	529587	USA
Lone Star	H	B	A	B	SA-0282	PI 528636	USA
Lou Mienne	B	B	A	B	SA-1423	PI 452103	China

Variety Name	CFB 5887	CFB 5888	CFBid 0001	CFBid 0002	SA#	PI#	Country of Origin
M240	A	H	A	A			USA
Magnolia	H	H	A	H	SA-0963	PI 529033	USA
Mar 5 PD208S-4-90	A	A	A	A	SA-2390	PI 603009	USA
Marico (Smooth)	H	H	A	A	SA-2330	PI 607197	S. Africa
McNair 210	A	A	A	A	SA-1535	529589	USA
McNair 235	A	A	A	A	SA-1472	529526	USA
MD 17	A	A	A	A			USA
MD 51ne	A	A	A	A			USA
MD 52ne	A	A	A	A			USA
Mebane	A	A	A	A	SA-0892	528985	USA
NM24016	A	A	A	A			USA
Pak-22 Vir-5850	A	A	A	A	SA-3232		Pakistan
Paymaster 101	A	A	A	A	SA-1021	PI 529090	USA
Paymaster 145	B	B	A	B	SA-1548	PI 529602	USA
Paymaster 303	A	A	A	A	SA-1551	PI 529605	USA
Paymaster 792	H	H	A	H	SA-1555	PI 529609	USA
PD-1	A	A	A	A	SA-1595	PI 606805	USA
PD-2	A	A	A	A	SA-1596	PI 606806	USA
PD-5256	A	A	A	B	SA-1736	PI 543865	USA
PD-93001(Brown)	A	A	A	A	SA-2210	PI 573281	USA
PD-93007	A	A	A	A	SA-2260	PI 591418	USA
PD-9364	A	A	A	A	SA-1575	PI 529629	USA
Pope	A	A	A	A	SA-0996	529065	USA
Pora INTA	A	A	A	A	SA-1762	PI 606820	Argentina
PSC355	A	A	A	A			USA
Reba P288	A	A	A	A	SA-2223	PI 607158	Chad
Rex	A	A	A	A	SA-1071	529140	USA
RN96527	A	A	A	A	SA-3492	PI 636103	USA
RN96625-1	H	H	A	H	SA-3493		USA
Rowden	H	B	A	B	SA-0300	PI 528649	USA
S-6524	A	A	A	A	SA-2572	PI 630195	Uzbekistan
Sabie	H	H	A	A	SA-2326	PI 607193	S. Africa
Saenz Pena 61	A	A	A	A	SA-0986	PI 529055	Argentina
Shan 5245	H	H	A	H	SA-3203	n/a	China
Shan 5710	H	H	A	A	SA-3204	n/a	China
Siokra 104-90	B	B	A	B	SA-2241	PI 607166	Australia
SL-7-1	A	A	A	A			USA
Stoneville	B	B	A	B	SA-1723	PI 547084	USA

Variety Name	CFB 5887	CFB 5888	CFBid 0001	CFBid 0002	SA#	PI#	Country of Origin
LA887							
Stoneville 2C	H	H	A	H	SA-0313	528659	USA
STONEVILLE 453	B	B	A	B	SA-3749		USA
Stoneville 474	H	A	A	A			USA
Stoneville 825	B	B	A	B			USA
Storm King	B	B	A	B	SA-1004	529073	USA
TAM 2562 RKNR	H	H	A	A	SA-2401	607225	USA
TAM 90J-57s	H	H	A	A	SA-2919	PI 614955	USA
TAM 98D102	A	A	A	A	SA-3516	PI 636490	USA
TAMCOT SP-21	H	H	A	B	SA-1580	529634	USA
TAMCOT SP-37	A	A	A	A	SA-1583	529637	USA
Tashkent I	H	H	A	B	SA-1392	PI 529447	USSR
Tashkent II	B	H	A	H	SA-1393	PI 529448	USSR
Tashkent III	H	H	A	H	SA-1394	PI 529449	USSR
Tejas	A	A	A	A	SA-2894	PI 591047	USA
TM-1	A	A	A	A			USA
UA48	B	B	A	B			USA
UK 64	A	A	A	A	SA-1285	PI 407455	Tanzania
Victoria	A	A	A	A	SA-1758	PI 606816	Spain
VIR-5817 LR-IV	B	B	A	B	SA-3237	n/a	Pakistan
VIR-6615 MCU-5	B	B	A	B	SA-3256	n/a	India
XA142 FUZZ	A	A	A	A			USA
XZ142 naked	B	B	A	B			USA
Zhong Mian Suo 7 hao	H	H	A	H	SA-3205	n/a	China
Zhong Mian Suo 8 hao	A	A	A	A	SA-3206	n/a	China
Zhong Mian Suo 9 hao	A	A	A	A	SA-3207	n/a	China
