# Supplementary material for: The Immature Fiber Mutant Phenotype of Cotton (Gossypium hirsutum) Is Linked to a 22-bp Frame-Shift Deletion in a Mitochondria Targeted Pentatricopeptide Repeat Gene
Source: G3 (Bethesda). 2016 Mar 29;6(6):1627–33. doi: 10.1534/g3.116.027649 (PMC4889659; doi:10.1534/g3.116.027649)
Supplement: Supplemental Material [file supp_g3.116.027649_TableS2.pdf]

Table S2 Primer Sequences

Name	Sequence
CFB5887_imF	GAATGATGTTCTCGTTTGTCTC
CFB5887_wtF	GAATGATGTTCTCGTTTGTCTA
CFB5887_R	ATTTCCCTGTTCTGATTTGTAA
CFB5888_wtF	AACACCCCTAAGTCATTTGTA
CFB5888_R	AAGGGACAACATGATTAGTTT
CFBid0001_F	TGCCTTCTCAATATCCCCTACT
CFBid0001_R	GGAAGGGCTATGAAGGGAACTA
CFBid0002_F	TTGAGGGCACGTCATTGATTA
CFBid0002_R	ACAAATATTTCCTCCTCTTTGGT
CFBid0003_F	TGAGGGGTTCTAGGTGCATA
CFBid0003_R	CGTGTGTTAAAGGTAAACATAGAGG
i00510Gh_imF	AAGGTTTGAATAGAAAACTGAG
i00510Gh_imR	TTTCGTTCGTACAAGAGG
i00510Gh_wtF	AAGGTTTGAATAGAAAACTGAA
i00510Gh_wtR	GCCCGTCGTTTCGTTC
Gh_A03G0468_F	TGTTTTGGCTAGTGAGGAATTG
Gh_A03G0468_R	ATCTTCAAACCTCCTTTGCCTG
Gh_A03G0469_F	AGTTGTTGCTCTCTTGGAAAAA
Gh_A03G0469_R	TAGTCCAGTAAATCGACGAACA
Gh_A03G0470_F	AGGTTGATGATGATGTTGGTTG
Gh_A03G0470_R	TTGGATATACTACACCGTCACC
Gh_A03G0471_F	ATCCTCCAAATGTTCCAGACAG
Gh_A03G0471_R	TCCATGGATATACCTATTGAGGAT
Gh_A03G0472_F	GCTTTGGGTGTCAAACTTATCA
Gh_A03G0472_R	ACTCGGGGATAGTAGTAAGAGT
Gh_A03G0473_F	TCCATGTTCTCCTGATCTTACC
Gh_A03G0473_R	CTACTAAGACTTCTAACCAATTTT
Gh_A03G0474_F	GATGACCATACTGAAGCTCAA
Gh_A03G0474_R	TGGAATCATCGATTTTGGGAAC
Gh_A03G0475_F	AGGTTGTACGAAAGTGCTATTTG
Gh_A03G0475_R	CCATTTATTCCTACCATCTGGGT
Gh_A03G0476_F	AGAGGTATGGGACCTTAATTGC
Gh_A03G0476_R	AAGGCTACATATTCCTCAGCTT
Gh_A03G0477_F	TCCAATCACCTTAATCCATCCA
Gh_A03G0477_R	GTTAATCTGTCTCCCTCGAGAA
Gh_A03G0478_F	CCATAGCACTGATGAGACGAAA
Gh_A03G0478_R	AATGAAGTTTGTAGCTTTGCCA
Gh_A03G0479_F	GCCCATATTCAGAAAGATGACG
Gh_A03G0479_R	CCATCAAGAACACCAACTGATT
Gh_A03G0480_F	AAGAATGGTGATATAGGTGCCA

Name	Sequence
Gh_A03G0480_R	TCAGAAGCACAAGTCTGTAATC
Gh_A03G0481_F	CGCTGTATCTAAACTTGCTGTT
Gh_A03G0481_R	ATTACCGGACCAACTTGTCATA
Gh_A03G0483_F	ATGGCAAAAGGTTCGGA
Gh_A03G0483_R	GGTCAACCTCTTGAGCAAAAA
Gh_A03G0484_F	TGCAAGACATTTCCAACATGAA
Gh_A03G0484_R	GTCATCATCGACCAAGACATTT
Gh_A03G0485_F	GCTGTTTAAGGTTTTTGACGTG
Gh_A03G0485_R	ACAATCTCTGATAATGTGCAGC
Gh_A03G0486_F	GAGCAGAAATCAATAAGTCCCG
Gh_A03G0486_R	CAATGGTAGTCTTTGCACTTGA
Gh_A03G0487_F	TGGTTGGTTGAAAACTGTTGAT
Gh_A03G0487_R	ATGCCTGTTCAACGTAAATGAA
Gh_A03G0488_F	TTTGTGTCATTGGCAGGAATTT
Gh_A03G0488_R	ATCCTCATGATCATCAACACCT
Gh_A03G0489_F	TATGCAAAAGAGAGGAGTTTTCG
Gh_A03G0489_R	GCTTGTGACATGTAATTGGACTG
Gh_A03G0490_F	CAAGCAGTCATTAGCTGTTTCAA
Gh_A03G0490_R	TATTCTCAGGATTGATGTCCTGC
Gh_A03G0491_F	CAAGTTGGAGGTGAAGGAGATA
Gh_A03G0491_R	CACCACCCTTTCCCTAAATCTT
Gh_A03G0492_F	CCTAATCAAAATCCACGTGCTG
Gh_A03G0492_R	CCGAAAACATACCTGCAGTAGTAA
Gh_A03G0493_F	TAGTCAAGGGGAGAGTGAAAATG
Gh_A03G0493_R	ATGTCCTTGTTCAAATCAACCTG
Gh_A03G0494_F	AGGAGTTTCTTCTTCTGCTTTGA
Gh_A03G0494_R	ACCGTTCGATGTTATCAAAAAGC
Gh_A03G0495_F	ACCCTTGATTCTCAAGCTGATG
Gh_A03G0495_R	CAGACTCTAGTGCATTTTGAAGG
Gh_A03G0496_F	GGTGTCTTCTAGGAGATTCGTTG
Gh_A03G0496_R	ACAAACCATTGTTATCTTCACGG
Gh_A03G0497_F	GCAGGAACTTATTCGGAAAGGAA
Gh_A03G0497_R	GAAAGGATGTATTGGCTTGAACC
Gh_A03G0498_F	GAGTCTAAGATGGTTATGCCTGG
Gh_A03G0498_R	CTTCTCTAATAGCAAACCGCATC
Gh_A03G0499_F	TCTCGTAATTGGACAAGTGTTGA
Gh_A03G0499_R	ACTCTGCTGAAATAAGGAGAAGC
Gh_A03G0500_F	AAATTCAGGGGACTTAAGAGGAG
Gh_A03G0500_R	GTACTTGAGCTCTTGCACTGATT
Gh_A03G0501_F	TGAGTGCTGCCTTGACAGATG
Gh_A03G0501_R	GTTCTTAACAACTTTGCTTACTTTCTGCC
Gh_A03G0502_F	GACTAAAGGGTTTGAGTCGTACA

Name	Sequence
Gh_A03G0502_R	CCTTGTATATTGGCCATTACCGT
Gh_A03G0503_F	CGATAGATGAGCTGAGAAAGTCA
Gh_A03G0503_R	GCTAACCCATGTCTGTATATCGT
Gh_A03G0504_F	ACCGTCTTCTTCAACTAAATGGA
Gh_A03G0504_R	CAGCAATAAGGCTATTAACCGTG
Gh_A03G0505_F	GAGATGGTTGTCTATAGTCCAGG
Gh_A03G0505_R	ATACTATACATGCTTCCCGCAAT
Gh_A03G0506_F	CTCACACAGGACCATCATATTCA
Gh_A03G0506_R	GACTAAGCTGAGAAGCAACAAAG
