# Supplementary material for: The Immature Fiber Mutant Phenotype of Cotton (Gossypium hirsutum) Is Linked to a 22-bp Frame-Shift Deletion in a Mitochondria Targeted Pentatricopeptide Repeat Gene
Source: G3 (Bethesda). 2016 Mar 29;6(6):1627–33. doi: 10.1534/g3.116.027649 (PMC4889659; doi:10.1534/g3.116.027649)
Supplement: Supplemental Material [file supp_g3.116.027649_TableS3.pdf]

Table S3 Domains of PPR Gh_ A03G0489 and nucleotide binding site position-specific weight matrix**.** Bolded residues are absent from either ORF in *im* transcripts. "1_4_ii" refer to the nucleotide specifying residues in the PPR repeats as in Yagi et al. 2013.

Motif	1_4_ii	A	C	G	T	Sequence
Transit Peptide						MRLSSKQTVYCFTKSRLSRLKSASLCTSASNDAEAVEIASILSKNDWKRLLETSSELT
linker-1						SKLNPEIVHSVLLQNSIHDPERLFSFFNWASQQSPNPQ
PPR-1	DSQ	0.362	0.131	0.205	0.302	NLDSFSFLAVTLCNSKMFRKATIVLDSMVRTRRPV
linker-2						QAILGSIIRCWKGYEGN
PPR-2	GDG	0.000	0.000	0.000	0.000	YVGVFDILIGCYKKMGSWNDAVYVFLGAKEVGVLP
PPR-3	AND	0.103	0.184	0.113	0.601	**GLACCNNFLGDLLKVNKLDLFWKVFNG**MIDAKLVP
PPR-4	YTG	0.466	0.090	0.284	0.160	DVYTFTNVINAHCRVGDIEKAKRVFMDMEEKGCSP
PPR-5	VND	0.078	0.308	0.034	0.580	GLVTYNVMIGGLCKAGAVDEALKLKNSMSRKGFNP
PPR-6	YNN	0.165	0.467	0.155	0.212	DAYTYNTVINGFCRHNRFQEAKLMMEEMRCAGLNP
PPR-7	FTN	0.755	0.069	0.057	0.120	NHFAYTALIDGFIKRGSMAEAFQVKDEMVACGIKP
PPR-8	FND	0.178	0.197	0.103	0.522	NVFTYNVLIGGASKAGDLEKGKALFDEMVLIGIGP
PPR-9	QST	0.362	0.131	0.205	0.302	DAQTFSLMIQSYCRQGNFLKAHELFEEMKEHNLTP
PPR-10	YNN	0.165	0.467	0.155	0.212	TMYTYNGIISGLCHCGDLGRANYVFEAMIKVGLKP
PPR-11	VTD	0.097	0.076	0.697	0.131	NVVMYTNLIKGHIQKSRFKEARKILNRMMENGQMA
PPR-12	FNN	0.449	0.076	0.344	0.131	DIFCCNTLLSGLCKSQMMDEARSFLVEMVDRGLKP
PPR-13	IGN	0.550	0.060	0.050	0.341	NEITYGTLIHGYAKAGEMEAVERCFREMRSYGIAP
PPR-14	ATD	0.162	0.104	0.586	0.149	NNAIYTILINSLCKVGNVTEALSTFRHMSEKGVLP
PPR-15	KTD	0.162	0.104	0.586	0.149	DIKTYTALIHGLAANGRINDAMQLFSQLDEKGIVP
PPR-16	FTN	0.755	0.069	0.057	0.120	DVFTYTSLISVFSKLGDMEAALNLYDKMCQKGIAP
PPR-17	VNN	0.211	0.609	0.047	0.133	NIVTYNAMIDGLCKLGDTEKARRVFNEIAQRGLAP
PPR-18	KSD	0.223	0.159	0.325	0.293	NVKSYSIIIDGYCKSGNLTEAFQLLDKMPSKGVPP
PPR-19	FCT	0.550	0.099	0.207	0.143	DSFTYCALIDGCCKGGELDKALSLFSEMLQKGFDP
PPR-20	SNN	0.165	0.467	0.155	0.212	-TSSFNALINGLCKTGKPNEAMRLLEDMASNCITP
PPR-21	RTN	0.573	0.106	0.147	0.175	NHRTYTILIDYHSKAGRMEEAEILFLEMQRRNLVP
PPR-22	VND	0.078	0.308	0.034	0.580	NVVTYNLLLHGYYRLGRKAEMFDLFESMAAKGVEP
PPR-23	IRD	0.000	0.000	0.000	0.000	DEIIYRLIANAYLEENSLIGMLKLLDEILVKDVVF
PPR-24	NFS	0.000	0.000	0.000	0.000	DKNPTFLLLDAVCKREEFSEVPKSLEEMAEQGLKL

Motif	1_4_ii	A	C	G	T	Sequence
PPR-25	IHD	0.000	0.000	0.000	0.000	SPITCHKLVHGFHDKGNPEKAEWILESLVRFGWIP
C-term						NTTTVNSIIDKENDVANLESPNNSPKQATCGVACQV
